# *Ophthatome*™: an integrated knowledgebase of ophthalmic diseases for translating vision research into the clinic

**DOI:** 10.1186/s12886-020-01705-5

**Published:** 2020-11-10

**Authors:** Praveen Raj, Sushma Tejwani, Dandayudhapani Sudha, B. Muthu Narayanan, Chandrasekar Thangapandi, Sankar Das, J. Somasekar, Susmithasane Mangalapudi, Durgesh Kumar, Narendra Pindipappanahalli, Rohit Shetty, Arkasubhra Ghosh, Govindasamy Kumaramanickavel, Amitabha Chaudhuri, Nagasamy Soumittra

**Affiliations:** 1 MedGenome Labs Limited, 3rd Floor, Narayana Nethralaya Building, Narayana Health City, #258/A, Bommasandra, Hosur Road, Bengaluru, Karnataka 560099 India; 2 Narayana Nethralaya, Bengaluru, India; 3 GROW lab, Narayana Nethrayala Foundation, Bengaluru, India; 4 MedGenome Inc, Foster City, USA

**Keywords:** Ophthatome, Knowledge base, Electronic medical records, Curated clinical data, Defined cohort, Vision sciences

## Abstract

**Background:**

Medical big data analytics has revolutionized the human healthcare system by introducing processes that facilitate rationale clinical decision making, predictive or prognostic modelling of the disease progression and management, disease surveillance, overall impact on public health and research. Although, the electronic medical records (EMR) system is the digital storehouse of rich medical data of a large patient cohort collected over many years, the data lack sufficient structure to be of clinical value for applying deep learning methods and advanced analytics to improve disease management at an individual patient level or for the discipline in general. *Ophthatome*™ captures data contained in retrospective electronic medical records between September 2012 and January 2018 to facilitate translational vision research through a knowledgebase of ophthalmic diseases.

**Methods:**

The electronic medical records data from Narayana Nethralaya ophthalmic hospital recorded in the MS-SQL database was mapped and programmatically transferred to MySQL. The captured data was manually curated to preserve data integrity and accuracy. The data was stored in MySQL database management system for ease of visualization, advanced search functions and other knowledgebase applications.

**Results:**

*Ophthatome*™ is a comprehensive and accurate knowledgebase of ophthalmic diseases containing curated clinical, treatment and imaging data of 581,466 ophthalmic subjects from the Indian population, recorded between September 2012 and January 2018. *Ophthatome*™ provides filters and Boolean searches with operators and modifiers that allow selection of specific cohorts covering 524 distinct ophthalmic disease types and 1800 disease sub-types across 35 different anatomical regions of the eye. The availability of longitudinal data for about 300,000 subjects provides additional opportunity to perform clinical research on disease progression and management including drug responses and management outcomes. The knowledgebase captures ophthalmic diseases in a genetically diverse population providing opportunity to study genetic and environmental factors contributing to or influencing ophthalmic diseases.

**Conclusion:**

*Ophthatome*™ will accelerate clinical, genomic, pharmacogenomic and advanced translational research in ophthalmology and vision sciences.

## Background

The field of medical records has evolved tremendously in the last century. However, the earliest history of clinical or medical record is about 4000 years old, an Egyptian case report, a surgical note dating back to 1600 BC. Hippocratic Corpus and other Greek scientific texts during the fifth Century BC and Medieval Islamic physicians’ case histories developed for didactic purposes are some of the early references available indicating practices of recording patient medical history. Detailed case books, recording symptoms and hospital records consisting admission and discharge books were followed by few physicians in the Europe and US in the mid-18th and early 19th centuries. The precedence for the modern medical records appeared in major centres in Paris and Berlin as loose paper files in the early nineteenth century. The system of assigning a clinical number and integrating all records of the patient into a single system to organize the scattered data was established by Henry S Plummer in 1907 at St. Mary’s hospital and Mayo clinic in the US. The inherent problems of paperwork and the advancement in the information and communication technologies (ICT) in the latter half of this century have propelled in an era of electronic medical records or electronic hospital records (EMR/EHR) [1]. The adoption of EMR has been faster and wider in the developed countries with countries like Canada, England, Germany, New Zealand achieving > 90% of the physicians or hospitals adopting EMR. The adoption of EMR in India is less compared to other developed countries and has been implemented by very few Government hospitals and large corporate hospitals chains including major tertiary care hospitals in the field of ophthalmology [2].

There are numerous ophthalmic registries in various countries, most of them being disease specific, like for cataract, glaucoma, age-related macular degeneration, corneal diseases, visual impairment and blindness, etc., which are either single centre or multicentric or regional or national or multinational or consortiums and the contribution being either mandatory or voluntary and in some of these the entry is through a web-based online portal [3]. The primary objectives of these registries are to understand the epidemiology of visual impairment and blindness, different diseases outcomes or adverse events associated with specific treatment or surgical interventions, thereby enabling improved patient care. The numerous disease specific registries across the globe record isolated data elements like demography, diagnosis, visual acuity, investigations such as (ocular coherence tomography (OCT), fundus fluorescein angiography (FFA)), management or complications, but not all these data elements are captured together in one unified registry [3].

The IRIS registry by the American Academy of Ophthalmology and French National Registry are two large databases built on data from EMRs [4, 5]. The two large databases, IRIS Registry (Intelligent Research in Sight) (https://www.aao.org/iris-registry/about) with 50 million patients data contributed by 14,793 physicians from 2937 practices (institutional and private) and the French National Registry, Système national d’information inter-régime de l’assurance maladie (SNIIR-AM) with 65 million patients data contributed by 1546 public and private healthcare facilities, both have comprehensive data extracted from EMRs and have organized the data in a searchable query format. The big data captured is analysed for clinical management improvement (both at individual patients’ level and assess practitioner’s standard of care), assess treatment outcomes, prevalence, and scientific research. However, no additional curation is performed on the data to ascertain for their data completeness, reliability, or validity [4, 5].

The EMR/EHR has been in place in India’s premier private ophthalmic institutes like Narayana Nethralaya (NN), Sankara Nethralaya (SN), LV Prasad Eye Institute (LVPEI) since the last decade. The eyeSMART EMR system of LVPEI connects 176 primary vision care centres, 18 secondary, 4 tertiary centres to a central hub for excellence and has captured 2,270,584 patient’s data over an eight-year period between 2010 and 2018 [6]. However, there is no published report yet on the system or volumes of data captured in their respective EMR from other ophthalmic institutes.

The EMR system is the digital storehouse of rich medical data that includes demographics, clinical information (diagnosis, clinical diagnostic tests, laboratory diagnostic tests, prescription treatment, surgical management,) and administrative (billing particulars including insurance) details of patients’ reviews, cross-references and visits to the hospital(s). Although EMR is a repository of vast clinical and health data on a large patient cohort collected over many years, the data lack sufficient structure to be of clinical value for applying deep learning methods and advanced analytics to improve disease management at an individual patient level or for the discipline in general. Therefore, aggregated data from hospital EMRs need to be captured in a structured knowledge base to support clinical and translational research (CTR) [7]. Medical big data analytics has revolutionized the human healthcare system by introducing processes that facilitate rationale clinical decision making, predictive or prognostic modelling of the disease progression and management, disease surveillance, impact on public health and research, and facilitating research collaboration.

MedGenome Labs Ltd., India, collaborated with Narayana Nethralaya (a tertiary care state of the art eyecare services) Bengaluru, India to build the *Ophthatome*™ - a knowledgebase of ophthalmic diseases covering over 500,000 individual electronic clinical records. The study describes methods and processes established for collecting high quality data, enriching the data with additional endophenotyping and functionalities and presentation of the data in a web-based portal that allows basic and advanced search functions. Finally, few case studies of the knowledgebase are given to highlight the clinical application, relevance and efficiency, and versatility of the *Ophthatome*™.

## Methods

### Data

*Ophthatome™* contains comprehensive clinical data captured from the EMR between September 2012 and January 2018 from two city centres of Narayana Nethralaya, a multi-speciality tertiary eye hospital in Bangalore, India. This dataset from the Indian population has complete clinical data of patient demography, disease specific attributes and treatment information recorded during each of the patient visits. The EMR data from Narayana Nethralaya recorded in the MS-SQL database was mapped and transferred to local MS-SQL. This was further programmatically transferred to MySQL. The captured data was manually curated, where missing data and ambiguities in the data were resolved by working with the clinician to preserve data integrity and accuracy. The data was stored in MySQL database management system for ease of retrieval, visualization, advanced search functions and other knowledgebase applications. The data is completely anonymised with unique identifier number and the privacy and confidentiality of the patient data is ensured. Written informed consent was obtained from the subjects or parents/legal guardians (in case when the subjects were < 18 years of age) at the time of registration at the hospital. The study is approved by institutional ethics committee of Narayana Nethralaya and adhered to the tenets of the Declaration of Helsinki.

### Data curation

**Fig. 1:**
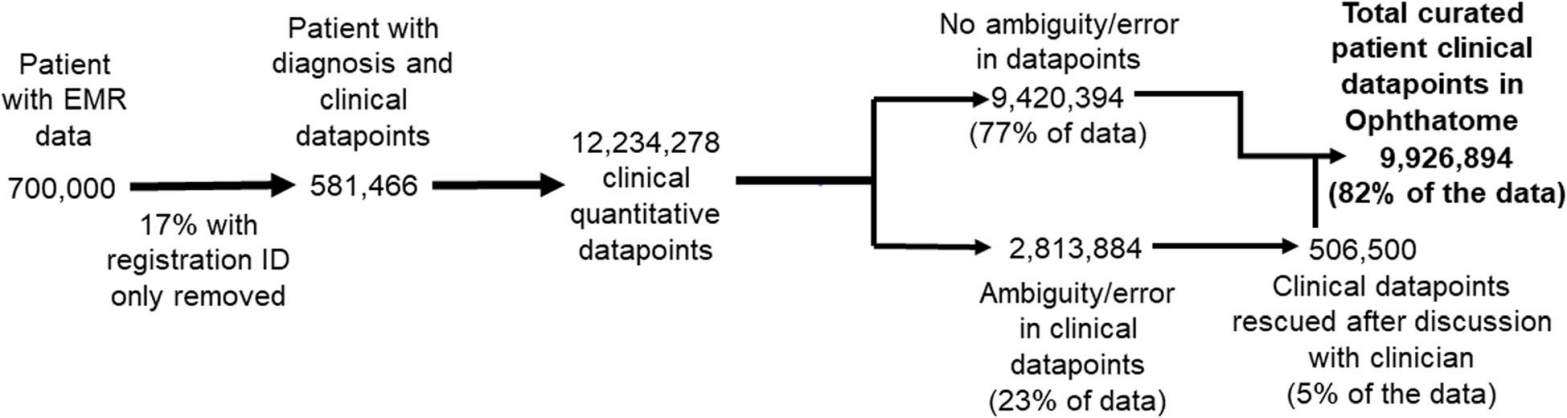
Shows the number of subjects with clinical diagnosis and the number of curated accurate datapoints in the *Ophthatome*™ knowledegebase

All demographic and clinical parameters for example, age, diagnosis, quantitative trait values were reviewed for validity and erroneous data was cleaned or flagged and removed. For each of the quantitative traits, the range of expected values were defined, and values falling outside the range were corrected after consultation with the clinician, or in case of ambiguity were deleted. Unstructured data, for example, disease diagnoses were reclassified using ICD10 code, personal history and family history of ophthalmic and systemic diseases were annotated with structured terminologies. *Ophthatome*™ is populated with extensively annotated data that were checked for accuracy and uniformity (Fig. 1). A brief overview of data types captured in *Ophthatome*™ are summarized below.

(i) Age
  The age was computed based on the date of birth and therefore reflects chronological age of subjects at multiple visits when longitudinal data is captured. Subjects with more than 110 years were marked as outliers, verified for accuracy of the data, and corrected. Paediatric age group included subjects ≤18 yrs [8] and subjects > 18 years were considered adult in the cohort.
(ii) Disease diagnosis
  The disease diagnosis in the EMR is based on ICD10 codes [9]. The ICD diagnosis was categorised into disease types and subtypes for comprehensive and informative querying of the data. For example, all types of cataract; cortical, nuclear, anterior subcapsular etc. were first grouped as cataract and then subclassified into their respective subtypes as shown in Table 1. Further, disease diagnoses entries that were not as per the ICD10 codes, or present in an unstructured format, or ‘free text’ in the EMR were assigned unique codes and grouped under appropriate disease types and subtypes. The unstructured disease diagnoses entries that specified two or more diseases, e.g., a) “primary open angle glaucoma (POAG) with cataract” or b) “early cataract with mild non-proliferative diabetic retinopathy (NPDR)” were grouped under both the diseases, a) glaucoma and cataract, and b) cataract and diabetic retinopathy and appropriate endophenotypes, respectively (Table 2). Each of the disease diagnosis was also annotated with additional data for example, (i) affected ocular organ (cornea, conjunctiva, retina etc), (ii) age of onset of the disease (as congenital, age-related, unspecified etc) and (iii) possible causes of the disease such as a) infectious (viral, bacterial, fungal or parasitic) b) genetic (complex, monogenic, somatic, mitochondrial, chromosomal) c) age-related d) developmental e) systemic causes f) secondary to other ocular diseases g) trauma i) related to eye surgery j) inflammation k) contact lens l) medications m) autoimmune disease n) environmental/allergy o) unknown. The categorization was done manually. These categorizations and labelling allow advanced search functions for identifying specific, well-defined cohorts and to raise specific queries and adding clinical richness to the database.
(iii) Quantitative traits
  - Refractive error (RE)
    Myopia and hyperopia were classified based on the degree as low, medium and high. The types of myopia are; low myopia (<− 3.00D), medium myopia (− 3.00D to − 6.00D) and high myopia (> − 6.00D) and hyperopia; low hyperopia (+ 2.00D), medium hyperopia (+ 2.25D to + 5.00D) and high hyperopia (> + 5.00D) based on the spherical power in diopters (D) [10, 11]. Entries other than the accepted integer in positive or negative values representing hyperopia and myopia respectively, were marked and removed.
  - Intraocular pressure
    Intraocular pressure (IOP) was classified as low, normal and high for the measurement values, < 12 mmHg, 12-21 mmHg and > 21 mmHg, respectively [12]. IOP values greater than 80 mmHg were considered erroneous and were excluded as maximum measurement that can be recorded on applanation and NCT (noncontact tonometer) are 80 and 60 mmHg, respectively.
  - Central corneal thickness
    Central corneal thickness (CCT) was defined as very thin, thin, average, thick and very thick for the values < 510 μm, 510-540 μm, 541-560 μm, 561-600 μm and > 600 μm respectively [13]. Values < 300 μm and > 700 μm were marked as outliers and corrected appropriately with reference to the longitudinal data if available or flagged and removed.
(iv) Systemic diseases
  The details of the systemic disease(s) were converted into standard terminology such as, diabetes mellitus, hypertension, coronary artery disease, renal stones, asthma etc. These labels improve search and selection functions in the web portal. The unique unstructured free text entries were exported to an excel file and for each entry (for the common diseases mentioned above), the corresponding standard terminology were assigned manually, which was later mapped in the database.
(v) Prescription drugs
  The EMR database contains a list of different prescribed ophthalmic and non-ophthalmic drugs as per their brand names. Generic names were appended for all the prescribed drugs along with their brand names. Further, the drugs were also grouped as per their therapeutic or pharmacological classification based on WHO/ATC classification. For combination drugs too, the generic, and the therapeutic or pharmacological classification were appropriately appended. This was done to identify cohorts which may not have been treated with the same generic or brand name drugs, but drugs with similar mechanism of action or pharmacological properties.
(vi) Family history
  Details of family history of disease(s) was available for 0.02% (12,465/561,466) of the cohort. The diseases were defined as either ophthalmic or systemic to choose subjects with family history of either or both type of disease(s). Here again the unique unstructured free text entries were exported to excel, the entries defined as ‘ophthalmic’ or ‘systemic’ disease manually and then mapped in the database.
(vii) Diagnostic procedural images
  The diagnostic procedural images that include, electroretinogram (ERG), fundus photograph, fundus fluorescein angiography (FFA), optical coherence tomography (OCT), frequency doubling technology perimetry (FDT), Heidelberg retina tomograph (HRT3), nerve fibre analysis (GDX), confocal microperimetry (MAIA), visual field analysis (HFA), Pentacam (anterior eye segment tomography), amplitude scan (ASCAN), specular microscopy, color vision, electrocardiograph (ECG), were all integrated into the *Ophthatome*™. The images along with the visit dates are presented as deidentified data retaining the same resolution as that of the original image.
  - Quantitative values within the procedural images
    The quantitative values like the visual field index (VFI) as percentage, median deviation (MD), pattern standard deviation (PSD) available in the visual field analysis image, average retinal nerve fibre layer (RNFL) thickness in micrometer, cup/disc ratio, available in the OCT image were programmatically read, and the values mapped and integrated in the database.
(viii) Visual impairment
  The subjects were defined either as normal vision or visually impaired based on latest unaided or best corrected visual acuity. First, the values in the unaided and best corrected visual acuity were classified as normal, visual impairment, severe visual impairment or blind based on standard WHO definition [14]. Subjects are defined normal if the unaided refraction is within normal limits. When the unaided refraction was classified either as visual impairment, severe visual impairment or blind, then the classification under the best corrected visual acuity of the better eye was considered to classify the visual impairment status of the individual.
(ix) Longitudinal data
  Approximately 50 % of the overall cohort in the knowledgebase have longitudinal data, i.e. have some clinical data recorded for more than one visit. In the *Ophthatome*™, for any visit > 1, an internal check of at least two datapoints for either refraction or glass prescription or slit-lamp examination was considered to short list cohort with longitudinal data.

**Table 1:** Categorization of diseases with ICD codes into disease types and subtypes for comprehensive and information querying in the *Ophthatome*™

ICD_code	Diagnosis_name	Disease	Sub_type
H25011	Cortical age-related cataract, right eye	Cataract	Cortical age-related cataract
H25012	Cortical age-related cataract, left eye	Cataract	Cortical age-related cataract
H25013	Cortical age-related cataract, bilateral	Cataract	Cortical age-related cataract
H25019	Cortical age-related cataract, unspecified eye	Cataract	Cortical age-related cataract
H25031	Anterior subcapsular polar age-related cataract, right eye	Cataract	Anterior subcapsular age-related cataract
H25032	Anterior subcapsular polar age-related cataract, left eye	Cataract	Anterior subcapsular age-related cataract
H25033	Anterior subcapsular polar age-related cataract, bilateral	Cataract	Anterior subcapsular age-related cataract
H25039	Anterior subcapsular polar age-related cataract, unspecified eye	Cataract	Anterior subcapsular age-related cataract
H2504	Posterior subcapsular cataract	Cataract	Posterior subcapsular cataract
H25041	Posterior subcapsular polar age-related cataract, right eye	Cataract	Posterior subcapsular age-related cataract
H25042	Posterior subcapsular polar age-related cataract, left eye	Cataract	Posterior subcapsular age-related cataract
H25043	Posterior subcapsular polar age-related cataract, bilateral	Cataract	Posterior subcapsular age-related cataract
H25049	Posterior subcapsular polar age-related cataract, unspecified eye	Cataract	Posterior subcapsular age-related cataract

**Table 2:** Categorization of unstructured disease diagnosis (non-ICD diagnosis) into disease types and subtypes for comprehensive and information querying in the *Ophthatome*™

ICD_code	Diagnosis_name	Disease	Sub_type
UK1486	Traumatic cataract LE	Cataract	Traumatic cataract
UK2123	Partially absorbed traumatic cataract	Cataract	Traumatic cataract

## Results

*Ophthatome™* contains clinical and phenotype data of 581,466 subjects (males - 318,383, females - 263,083) with 524 distinct ophthalmic disease types and 1800 disease sub-types.

**Fig. 2:**
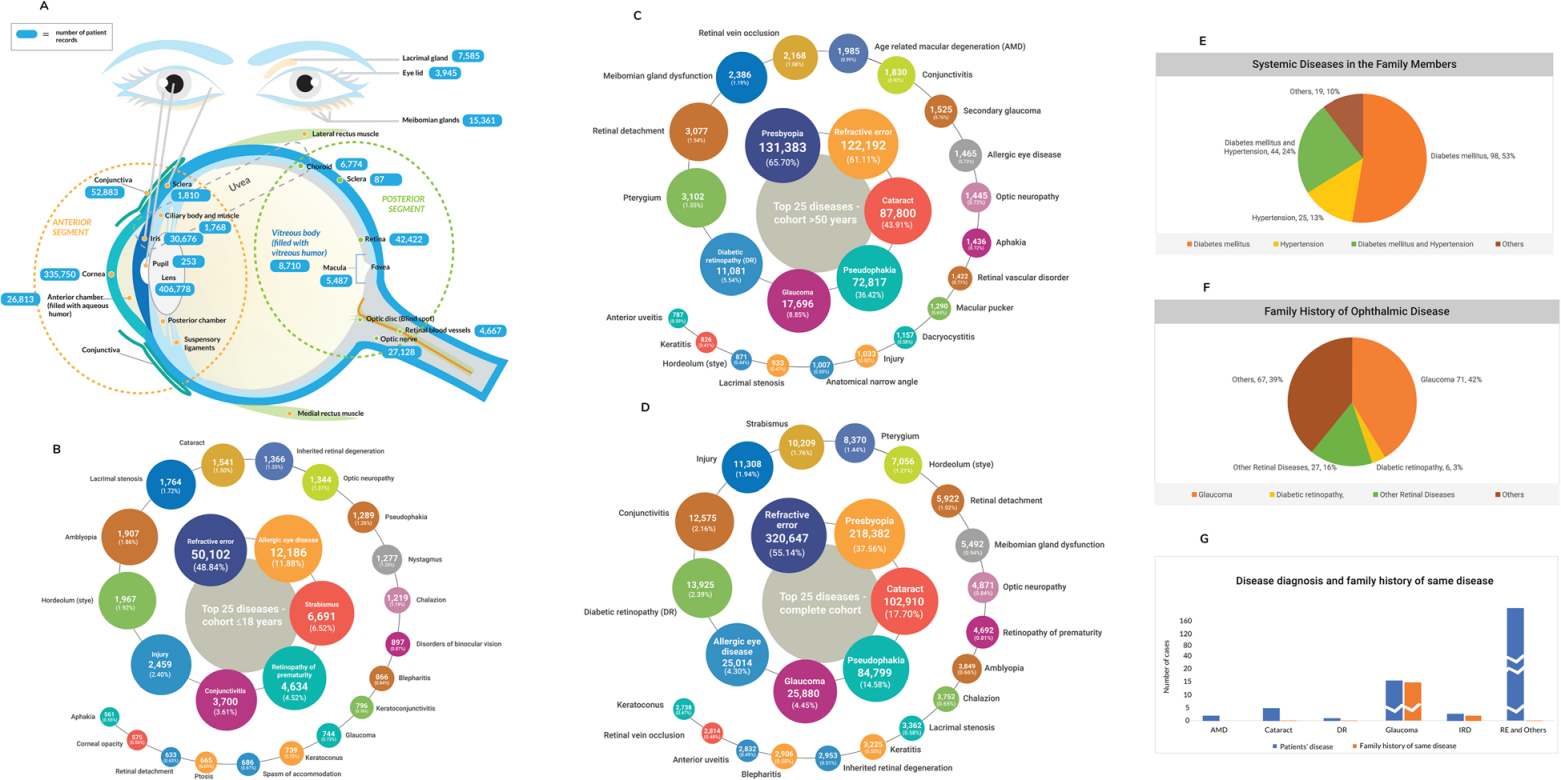
**a** Summarizes the number of patient data available for various diseases affecting different parts of the eye in the database. **b** Shows the top 25 ophthalmic diseases of the paediatric subjects (≤ 18 years) in the database. **c** Shows the top 25 ophthalmic diseases of the > 50 years cohort in the database. **d** Shows the top 25 ophthalmic diseases of the overall cohort in the database. **e** Represents common systemic diseases of the 186 subjects with family history of both ophthalmic and systemic diseases. **f** Presents details of family history of ophthalmic diseases of the 186 subjects with family history of both ophthalmic and systemic diseases. **g** Shows the details of ophthalmic disease and family history of same disease in the 186 subjects with family history of both ophthalmic and systemic diseases

The knowledgebase comprises diseases affecting 35 different ophthalmic anatomical parts including the orbit, eyelids, anterior segment, posterior segment, and the ocular adnexa. Figure 2a summarizes the number of patient data available for various diseases affecting different parts of the eye. In summary the knowledgebase includes comprehensive ophthalmic clinical variables such as refraction, intraocular pressure, central corneal thickness, slit-lamp examination details, diagnosis, prescription medications, surgical interventions, and thirteen clinical diagnostic images. The available clinical variables for each subject were mapped longitudinally starting with the most recent value. *Ophthatome™* is unique, clinically rich, and versatile as it contains millions of curated quantitative trait datapoints. Of the total of 12,234,278 quantitative datapoints (RE, IOP, CCT), 9,240,394 (77%) were within the defined range of value for the trait. Manual curation added 506,500 (5%) datapoints resulting in a total of 9,926,894 (82%) accurate datapoints (Fig. 1).

### Analysis of age-related ocular diseases

The top 25 ophthalmic diseases affecting < 18 years (paediatric subjects), > 50 years and for the overall cohort are given in Fig. 2b, c, d respectively. A comparison between the two age groups, show that refractive error is equally prevalent in both the groups (46,569/102,590 paediatric subjects and 271,147/478,876 subjects in the > 18 years age group), whereas presbyopia, which is a condition associated with age was completely absent in ≤18 years old and is diagnosed in 217,765 subjects > 18 years age. Similarly, late-onset eye disease, like age-related macular degeneration was completely absent in the paediatric group as expected and is reported in 2166 subjects of adult population. Conversely, retinopathy of prematurity and retinoblastoma was reported in the paediatric group only (4695 and 213 subjects, respectively) and completely absent in the adult. Cataract is reported in both age groups (3511/102,590 of ≤18 years and 187,314/478,876 of > 18 years), but the prevalence is higher in the > 18 years cohort reiterating that congenital form is rare compared to commonly present age-related cataract in a population. The same was observed with glaucoma as well (813 subjects in ≤18 years and 25,485 subjects in > 18 years). Further, the prevalence of primary angle closure glaucoma (PACG) (2983 of the 25,485 (11.7%) subjects in > 18 years age) is almost similar to primary open angle glaucoma (POAG) (2763/25,485 (10.8%)) in our cohort which is in accordance with the fact that prevalence of PACG is higher in Asians compared to the Caucasians. The clinical data summary of the overall cohort is presented in Table 3.

**Table 3:** Clinical data summary of the overall cohort

Clinical parameters information available (at least one datapoint)	Number of subjects
Diagnosis	568,324
Refraction	534,253
Intraocular pressure	520,874
Central corneal thickness	11,460
Systemic history	239,966
Medications prescribed	231,223
Ophthalmic surgery performed	134,686

*Ophthatome*™ contains 5777 (0.99%) subjects reporting family history of certain ophthalmic disease, 7010 (1.20%) subjects reporting a family history of systemic disease(s) and 186 subjects (0.03%) reporting family history of both ophthalmic and systemic diseases. Fig. 2e, f and g represents the details of systemic diseases of the family members (first or second degree relative or spouse), family history of ophthalmic diseases and disease diagnosis and family history of same disease, respectively in the 186 subjects with family history of both systemic and ophthalmic diseases. Fig. 2g shows that among all the common eye diseases, glaucoma and inherited retinal degenerations have a genetic component as expected.

### Research and clinical applications of *Ophthatome*™

#### Cohort selection

Complex diseases present with various analytical challenges, including lack of clinical homogeneity in disease phenotype, variability in disease progression rate, response to drugs and disease outcome. Therefore, aggregation of clinical data from large patient cohorts are needed to identify common as well as divergent trends in disease progression. *Ophthatome*™ provides many strategies for subject selection through filters and Boolean searches with operators and modifiers. Selection of cohorts based on (i) demographics, (ii) disease type and subtype, (iii) quantitative traits like a) refractive error (myopia and hyperopia), b) intraocular pressure, c) central corneal thickness, (iv) number of visits (v) systemic diseases (vi) family history of ophthalmic and/or systemic diseases (vii) diagnostic procedural images (viii) visual field index values (ix) visual impairment status and (x) drugs prescribed, are available with the ability to build complex multiparametric searches.

**Fig. 3:**
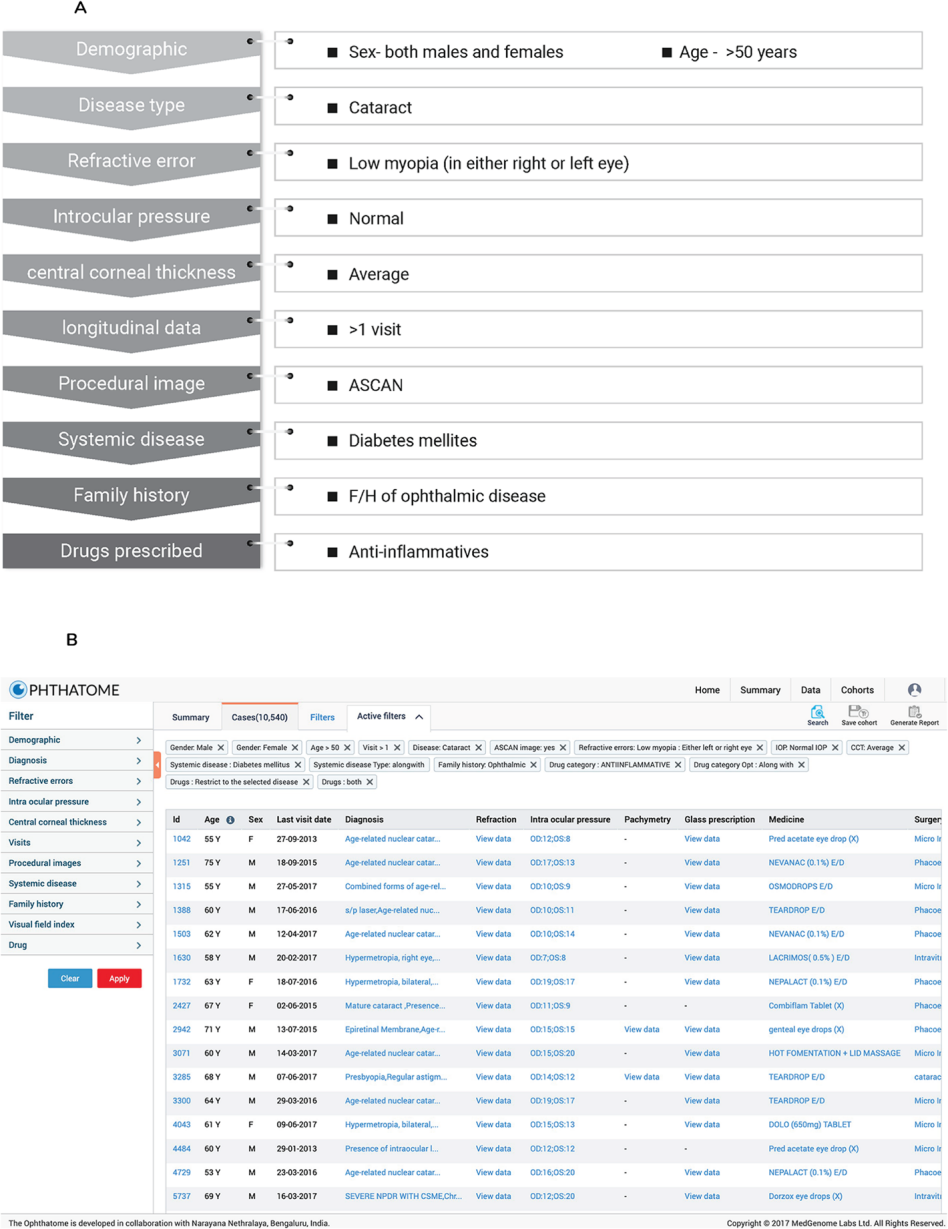
**a** Represents a flow chart of choosing filters successively to obtain a specific cohort. **b** Is the snapshot of specific cohort shortlisted after selecting filters successively as shown in **a**

A series of filters covering demography, clinical features and prescribed drugs can be applied in *Ophthatome*™ (Fig. 3a) to select a cohort of patient samples (10,540 subjects) as represented in Fig. 3b. This number can be further narrowed using additional filters to create a near homogeneous cohort sharing specific disease phenotypes for clinical or genetic analyses, for example.

#### Cohort selection for deeper analysis

Few case studies are as presented here.

**Fig. 4:**
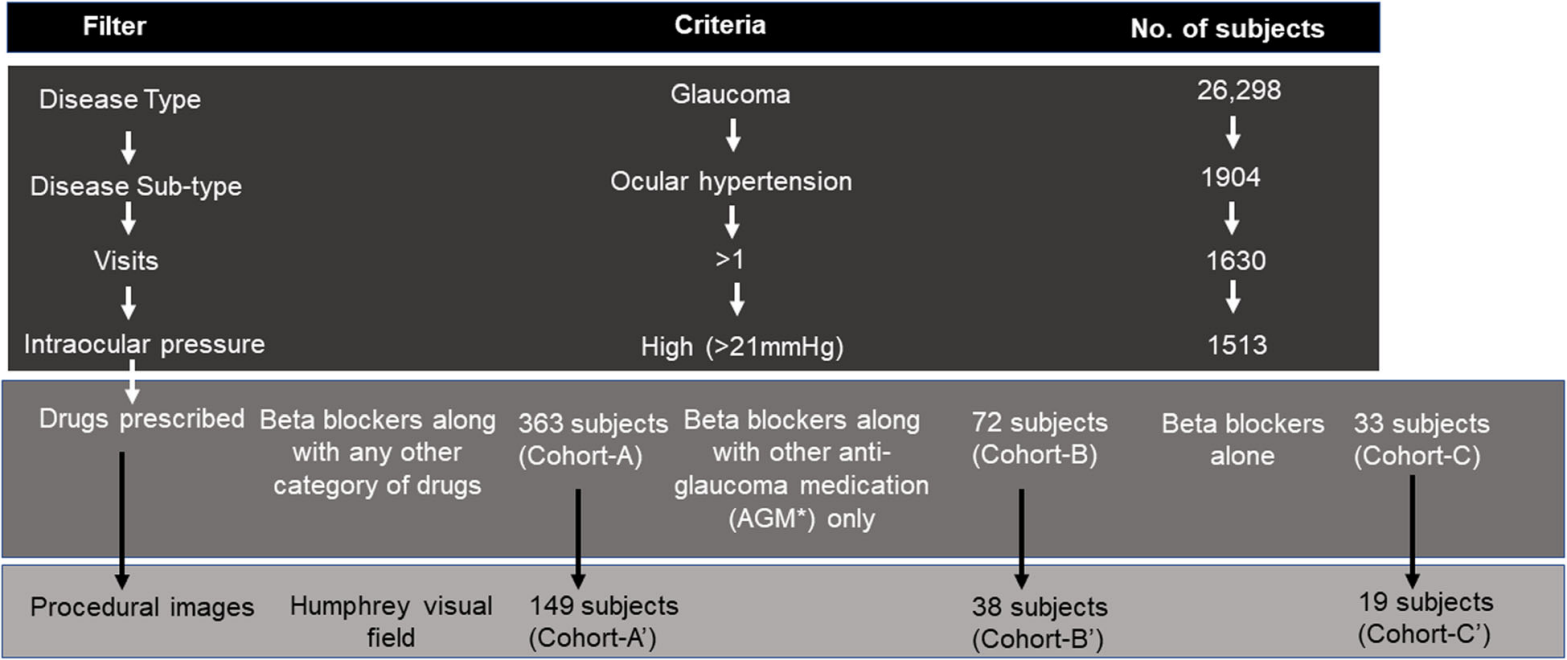
Shows combination of filters used, and the cohorts obtained that would help to study and understand the response of beta blockers alone or in combination with other anti-glaucoma (AGM) drugs in subjects with ocular hypertension

Glaucoma is a heterogeneous disease with multiple subtypes, and with a myriad of treatment/management options. To compare patient’s response to beta-blockers alone, or in combination with other anti-glaucoma medications, a combination of filters was used to select appropriate disease cohorts as shown in Fig. 4. Glaucoma used as a disease type and ocular hypertension used as the disease subtype, shortlists 26,298 (4.52% of the entire cohort) and 1904 subjects (0.32%), respectively. Additional filters applied successively include, > 1 visit – 1630 subjects, high intraocular pressure – 1513 subjects, drugs prescribed; cohort-A, beta blockers + other category of drugs – 363 subjects, cohort-B, beta blockers + other anti-glaucoma medication (AGM) – 72 subjects, and cohort-C, beta blockers alone – 33 subjects. Availability of Humphrey visual field image for each of the three cohorts further narrowed the numbers as shown in Fig. 4. The comprehensive clinical details of each patient can be extracted in a tabular format from the database and used for further analytics. Follow up data are available for a subset of patients under different treatment conditions and analysing subjects with at least two VFI datapoints at ≥ one-year interval (Table 4) revealed number of subjects that were stable and those who progressed. For example, of the 9 individuals treated with beta blockers alone, 7 had stable VFI and 2 converted to primary angle closure (PAC) within 2 years. Additionally, of the 43 patients treated with beta blockers + AGM,11 subjects were not further analysed as they were diagnosed with either POAG or secondary glaucoma in the first visit along with OHT. However, 29 of the remaining 32 treated patients had stable VFI for 4 years, one subject converted to PAC and two individuals progressed to POAG, in 4 years. Cumulative data from drug responsive and non-responsive patient groups can be further investigated using molecular and genomic tools.

**Table 4:** Number of subjects treated with beta blocker alone or along with other AGM and at least 2 VFI datapoints at > 1 year interval

# of subjects prescribed with BB only and > 1 year interval between the VFI datapoints	9
# subjects prescribed with BB and other AGM and > 1 year interval between the VFI datapoints	43

Drugs blocking growth of new blood vessels (neoangiogenesis) has improved vision dramatically in a large subset of patients with diabetic retinopathy and wet age-related macular degeneration (wet-AMD). *Ophthatome*™ has information of 5466 patients treated with anti-angiogenic therapy such as *Avastin* and *Lucentis* (Table 5). Follow up data on these individuals have identified drug resistance in a fraction of the treated patients opening opportunities to understand the mechanism of resistance (Unpublished data).

**Table 5:** Diseases and number of subjects treated with different anti-angiogenic drugs

Anti-VEGF molecules	Avastin	Lucentis	Accentrix	Eylea	Razumab	Anti-VEGF (not specified)
Total number of subjects	3706	942	860	35	310	279
Diseases wise distribution						
Wet AMD	60	17	23	3	7	5
AMD	102	36	38	8	17	10
Diabetic retinopathy	1272	256	190	_	65	75
Retinal vein occlusion	496	122	119	4	28	26
Myopic CNMV	4	1	_	_	_	_
Pathological myopia	5	3	1	_	1	1
Neovascular glaucoma	199	37	27	1	10	12
Retinopathy of prematurity	2	_	_	_	_	_
Retinal vascular disorder	579	174	65	10	18	21
Retinal vasculitis	20	3	2	_	_	1
Chorioretinopathy	20	7	4	1	1	3

Seasonal allergen or seasonal changes show increased incidence of infectious or non-infectious conjunctivitis. *Ophthatome*™ has 12,594 subjects of conjunctivitis and the subtype classification reveal 7414 subjects of viral, 1604 bacterial and 912 allergic conjunctivitis. Analysing the time period of highest incidence of infection or allergy would reveal details of epidemiological trends in disease outbreak, the causative infective agent or the appearance of seasonal allergens trends that could be used for treatment, prophylactic disease management, public awareness and preventive strategies.

## Discussion

Comprehensive and curated phenotype data captured over many years provide a wealth of quantitative and qualitative clinical parameters to perform in-depth phenotype analysis, disease progression/prognosis, severity of disease and variations in response to therapeutic measures. *Ophthatome*™ is a unique database that captures ophthalmic diseases from a genetically heterogeneous population in India.

There are a few reports published globally from the EMR based big data registries establishing and reiterating the need for such databases. From the IRIS registry - A study to understand the incidence of post cataract surgery endophthalmitis identified the incidence as 0.04% for cataract surgery performed on 8,542,838 eyes (5,401,686 patients) between 2013 and 2017. Younger age and cataract surgery along with other ophthalmic surgery and anterior vitrectomy was found to be risk factors [15]. In another study to assess the real world management of diabetic macular edema (DME), it was observed that subjects presented with better visual acuity (VA) at presentation received no treatment but those presented with lower VA at index date were promptly treated with anti-VEGF which improved the VA at end of 1 year, and further patients who received > 5 injections per year had a better VA gain compared to those who received 1–5 injections during the same period [16]. In yet another analysis from the IRIS registry study, performance rate measurements with feedback to physicians and administrators had improved subsequent performance [17]. The two publications from the EPISAFE programme on the congenital cataract surgery and cataract surgery report the incidence of congenital cataract between 2010 and 2012 to be 1–3/1000 birth, same as the other industrial nations, while the incidence of cataract surgery (2,717,203 eyes and 1,817,865 patients) increased from 9.86–11.08/1000-year between 2009 and 2012. The incidence of retinal detachment (RD) and pseudophakic cystoid macular edema being 0.99 and 0.95%, respectively with higher risk of RD for patients < 60 years of age [5, 18].

Two reports from the eyeSmart database, currently the only published EMR based bigdata database from India reveal incidence of dry eye disease (DED) and allergic eye disease (AED) in the population studied between the years 2010 and 2018.

Of the 1,458,830 new patients recruited 21,290 (1.46%) had recent-onset DED with age, gender, residence, occupation, socio-economic status having a significant impact in development of the disease [19], while of the 259,969 new patients (≤21 years) 10.1% had AED. Boys with atopy during mid childhood and from middle to higher income families were commonly affected by AED which was self-limiting by adolescence [20].

The above reports present the real-world clinical practice and their outcomes that provide information and details on incidence, trends, risk factors, treatment outcomes, prophylaxis, and quality assessment.

*Ophthatome*™ though currently has > 0.5 million patient records, the data is captured from a single hospital contributed by more than 100 ophthalmologists. The data was aggregated using a streamlined and standardized format using uniform clinical terms across departments and sub-specialities in the organization, thereby avoiding ambiguity, which may arise if data is captured across many different medical facilities as is the case with other large registries. As described in the Results section, despite uniformity of data capture, manual curation of the captured data revealed lack of consistency in 23% of the data. This was resolved in further 5% through discussion with the clinicians resulting in 82% of the data being accurate. The public registries driven by clinician-reported clinical and phenotype data are most likely to contain inaccuracies and inconsistencies, which are difficult to resolve in a short time, given the size of the data and the scope of the registries and differences in annotation practices.

Yet another limitation of the large registries like the IRIS is the unavailability of the diagnostic images. In the study presenting the real – world management of DME, the data on number of DME subjects is extracted based on ICD, where the ophthalmologist may have defined the case with the given code for further follow up and eligibility and does not indicate confirmed diagnosis. It is not possible to assess the severity of the disease in the absence of imaging data and thus the percent of patients managed with intervention and observation only and the outcome needs to be understood and interpreted with caution [16]. *Ophthatome*™ integrates diagnostic images along with the quantitative values within the image programmatically captured and mapped in the database. This adds much value to the knowledgebase as it helps to further confirm the disease severity and suitability of the chosen cohort for further analysis.

*Ophthatome*™ is a licensed database with patient-consented data which enables users to build highly specific disease cohorts using combined search terms and built in logical functions for research in ophthalmology and vision sciences.

*Ophthatome*™ will be updated on a regular basis to capture new data for existing entries in the database as well as new patient data, though currently it is not linked real-time.

## Conclusions

*Ophthatome*™ represents a versatile curated database of over 1800 ophthalmic diseases with longitudinal data associated with disease outcomes, drug responses and secondary complications. It also provides opportunity to design genomic and pharmacogenomic studies that would help understand the biology of complex ophthalmic diseases and is a powerful tool for advanced research in ophthalmology and vision sciences, including phase IV studies in an unique population and region.

### Link for demo version

https://demo.ophthatome.com/

## Data Availability

The datasets generated and/or analysed during the current study are not publicly available due confidentiality of the patients’ clinical data but an anonymized form of the datasets generated and/or analysed during the current study are available from the licensed version of the *Ophthatome*™ knowledgebase upon reasonable request.

## Abbreviations

CCT: Central corneal thickness
CTR: Clinical and translational research
ECG: Electrocardiograph
EMR: Electronic medical records
ERG: Electroretinogram
FFA: Fundus fluoresce angiography
IOP: Intra ocular pressure
MD: Median deviation
MSVI: Moderate to severe visual impairment
NPDR: Non-proliferative diabetic retinopathy
OCT: Optical coherence tomography
PSD: Pattern standard deviation
PACG: Primary angle closure glaucoma
POAG: Primary open angle glaucoma
RNFL: Retinal nerve fibre layer
VFI: Visual field index
